# Associations among diet, gut microbiota, and hypertension: a cross-sectional study in Thai subjects

**DOI:** 10.7717/peerj.21135

**Published:** 2026-04-21

**Authors:** Phatthanaphong Therdtatha, Thanapoj Buakhao, Niwed Kullawong, Vasana Jinatham, Thanakrit Vichasilp, Jiro Nakayama, Siam Popluechai

**Affiliations:** 1 Specialized Research in Microbiome and Metabolome for Health Laboratory, Division of Biotechnology, Faculty of Agro-Industry, Chiang Mai University, Chiang Mai, Thailand; 2 Department of Biochemistry, Phramongkutklao College of Medicine, Bangkok, Thailand; 3 Gut Microbiome Research Group, Mae Fah Luang University, Chiang Rai, Thailand; 4 School of Health Science, Mae Fah Luang University, Chiang Rai, Thailand; 5 School of Science, Mae Fah Luang University, Chiang Rai, Thailand; 6 Laboratory of Microbial Technology, Division of Applied Molecular Microbiology and Biomass Chemistry, Department of Bioscience and Biotechnology, Faculty of Agriculture, Kyushu University, Fukuoka, Japan

**Keywords:** Hypertension, Gut microbiota, *Phascolarctobacterium*, Alistipes, Cardiovascular diseases

## Abstract

**Background:** Although the link between the gut microbiota and hypertension has been investigated, its specific role in the increasing prevalence of this disease in Thailand, influenced by changing dietary patterns, remains unexplored. This cohort study investigated the association between the gut microbiome and hypertension-related dietary habits in Thai subjects.

**Methods:** The fecal microbiomes of 31 Thai adults, including non-hypertensive (NHT, *n* = 12) and hypertensive (HT, *n* = 19) subjects, were grouped and analyzed according to their dietary and medical records. Alpha and beta diversity analyses were performed to determine significant differences (*p* < 0.05) in microbial diversity between groups. Variations in the microbiome profiles were identified using Linear Discriminant Analysis Effect Size (LEfSe) based on a linear discriminant analysis score > 2.0 (*p* < 0.05). Multiple Factor Analysis (MFA) was employed to integrate anthropometric data, dietary consumption, and gut microbiome, enabling the visualization of features driving overall variation. Functional profiles of the gut microbiome were predicted using PICRUSt2 based on the Kyoto Encyclopedia of Genes and Genomes (KEGG) categories. Differential abundance and microbial-functional associations were evaluated using ALDEx2 and HAllA, respectively.

**Results:** Our exploratory analysis suggests that hypertension-related differences are strongly associated with host factors (age and clinical profiles) rather than diet or microbial composition. Key taxonomic signatures of the HT group include the expansion of *Phascolarctobacterium* and depletion of *Alistipes*, which relates to anthropometric and blood profiles. Functional analysis revealed a profound restructuring of the gut ecosystem in the HT group, characterized by functional dysbiosis where microbial-functional association patterns shift distinct from changes abundance.

**Conclusions:** These findings suggest that the gut microbiota’s potential role in hypertension may involve altered interaction dynamics, which may provide a new perspective for targeted interventions in the Thai population.

## Introduction

High blood pressure (BP), also known as hypertension, is considered the main risk factor for metabolic diseases, with more than 85% of patients showing obesity and type 2 diabetes (T2D) comorbidity (Franklin, 2006; Tanaka & Itoh, 2019). Several factors are associated with the progression of hypertension. Dietary habits profoundly influence hypertension (Zhao et al., 2011). Excessive consumption of saturated and trans fatty acids (Yuan et al., 2020; Gaeini, Bahadoran & Mirmiran, 2022; Li et al., 2022a), high sodium intake (Grillo et al., 2019), and excessive sugar consumption (Mansoori et al., 2019; Zhao et al., 2023) have all been directly implicated in increasing BP. In addition, the gut microbiota is an important indirect pathway that links diet and hypertension. For instance, the consumption of red and highly processed meat, which is rich in precursors such as choline, carnitine, and lecithin, can be metabolized by gut bacteria into trimethylamine (TMA), which the liver converts to trimethylamine N-oxide (TMAO), a metabolite that may impair blood vessel function and potentially lead to higher blood pressure (Verhaar et al., 2020). Understanding the direct and gut-mediated pathways through which diet influences hypertension is crucial to address this prevalent condition.

Asia has a particularly high burden of hypertension. Previous studies have suggested an overall prevalence ranging from 15–35% in urban adult populations based on the latest diagnostic criteria (140/90 mmHg) (Kang et al., 2019; Wang et al., 2021; Zhang et al., 2023). This value is considerably higher than the global average. Rural areas tend to have a lower prevalence, but the gap is narrowing because of factors such as urbanization and lifestyle changes. Thailand, a Southeast Asian nation, reflects regional trends in hypertension prevalence. Previous studies have indicated an increasing prevalence, particularly in urban areas (Thawornchaisit et al., 2013; Sakboonyarat et al., 2023; Sakboonyarat & Rangsin, 2024). Factors such as unhealthy dietary patterns, high salt intake, increased obesity rates, and physical inactivity, are also significant contributors. Additionally, the aging population and increasing stress levels further exacerbate this problem.

The human gastrointestinal (GI) tract harbors a vast, diverse community known as the gut microbiota, comprising approximately 100 trillion microorganisms, primarily bacteria, viruses, fungi, and protozoans. This intricate ecosystem is essential for digestion, nutrient absorption, and immune functions (Thursby & Jude, 2017). Emerging evidence suggests that imbalances in the gut microbiota composition may be associated with various health conditions, including hypertension (Tanaka & Itoh, 2019; Yang et al., 2023). However, the exact mechanisms by which the gut microbiota influences BP are still under investigation. Gut bacteria that produce lipopolysaccharides, such as *Escherichia coli* can trigger inflammation and contribute to hypertension (Grylls, Seidler & Neil, 2021). Studies in animal models have reported that high salt intake leads to an imbalance in the gut bacterial community, increasing the abundance of Lachnospiraceae, *Ruminococcus*, and *Parasutterella*, but decreasing the abundance of *Lactobacillus* and *Oscillibacter* (Miranda et al., 2016; Bier et al., 2018). Human studies, although fewer and often smaller than those using animal models, have shown preliminary associations between altered gut microbiota (specifically, *Lactobacillus* depletion), immune system changes (such as TH17 activation), pro-inflammatory states, and increased blood pressure (Wilck et al., 2017). Certain gut bacterial families, particularly Clostridia and Enterobacteriaceae, break down dietary nutrients, such as carnitine, choline, and lecithin, found in animal products. This breakdown process can lead to the production of TMA, a precursor converted to TMAO in the liver (Verhaar et al., 2020).

Hypertension is a major public health concern in Thailand. While previous studies have explored the potential link between the gut microbiota and hypertension globally, data within the Thai population are limited, especially in rural areas where traditional lifestyles and dietary patterns may differ significantly from those in urban settings. This pilot study aimed to investigate the association between gut microbiome composition and dietary patterns, both of which are potential influencing factors for the development of hypertension, in Chachoengsao Province, a developing city in Thailand. Additionally, we aimed to capture the gut microbiome status of hypertensive patients and compare it to that of healthy individuals. Investigating the features of the gut microbiome associated with diet and hypertension in the Thai population may be valuable for future research to develop targeted prevention, dietary, and microbiome management strategies.

## Materials and Methods

### Ethics declaration

This study was conducted in accordance with the guidelines of the Declaration of Helsinki and approved by the Ethics Committee of the Institutional Review Board of the Royal Thai Army Medical Department (IRB numbers: IRBRTA0320/2024). All methods were performed in accordance with relevant guidelines and regulations. Written informed consent was obtained from all study participants. Samples and questionnaire data were entered and analyzed anonymously, and all data were published anonymously using numbers to represent the subjects.

### Study design

Thai adult subjects aged 20–70 years old, who lived in Sanam Chai Khet district, Chachoengsao Province (location: 13°39′29.7″N 101°26′19.9″E), were targeted through community clinics in the province. Subject screening was performed based on inclusion and exclusion criteria (Data S1). The hypertensive group was classified based on a previous diagnosis and hypertensive severity determined by physicians according to the 2020 International Society of Hypertension Global Hypertension (ISH) Practice Guidelines (Unger et al., 2020) and the 2021 World Health Organization (WHO) guidelines on the pharmacological treatment of hypertension (Campbell et al., 2022). Participants who met the inclusion criteria were further involved in the activities of the study by completing the baseline questionnaire and having their personal physiological information, including age, sex, weight, height, body mass index (BMI), and medication history (Table S1), recorded by a medical technologist. A monthly dietary questionnaire modified from the questionnaire reported by Chumponsuk et al. (2021) was used to interview the participants about the weekly frequency of their consumption of common Thai foods and drinks (Table S1). The questionnaire comprised 18 questions that categorized participants across eight dietary behavior categories: carbohydrate consumption (nood_w: noodle; sticr_w: sticky rice; bread_w: bread), grain consumption (grain_w: grains; bean_w: bean; brice_w: brown rice), protein consumption (fish_w: fish; egg_w: egg; milk_w: fresh milk), vegetable and fruit consumption (vetf_w: vegetables and fruit), high-sugar food and beverage consumption (fruitj_w: fruit juice; cola_w: cola or carbonated drinks; teacof_w: tea and coffee), high-salt food consumption (fruitd_w: pickled fruit), high-fat food consumption (fat_w: high fat diet), and processed food consumption (pork_w: pork; chic_w: chicken; beef_w: beef). Differences in the mean ranks of food items between the hypertensive (HT) and non-hypertensive (NHT) groups were assessed using a Wilcoxon rank-sum test with a significance threshold of *p* < 0.05.

Clinical data for blood cholesterol and triglyceride levels were collected and measured by medical technologists using the cholesterol oxidase/peroxidase (CHOD/POD) and glycerol-3-phosphate oxidase/peroxidase (GOP/POD) methods, following the manufacturers’ protocols. Blood pressure was measured thrice using a mercury sphygmomanometer. The subjects were asked to collect their fecal samples using sampling kits provided by the researchers. Ultimately, 31 patients were included in the study. The 31 participants were classified into the HT and NHT groups based on a blood pressure cutoff of ≥140 mmHg for systolic blood pressure (SBP) and ≥90 mmHg for diastolic blood pressure (DBP), as defined by the ISH and WHO Practice Guidelines (Unger et al., 2020; Campbell et al., 2022). The demographic and clinical characteristics of the 31 patients are summarized in Table 1.

**Table 1:** Demographic and clinical characteristics of 31 Thai subjects in this study. Values are presented as mean ± standard deviation, except for gender.

Variable	NHT (*n* = 12)	HT (*n* = 19)	Significant level	Statistical test
Gender (male/female)	2 /10	8/11	*p* = 0.24	Fisher’s Exact Test
Age	42.25 ± 12.74	53.32 ± 5.55	*p* = 0.01	Welch’s t-test
Weight (kg)	60.54 ± 11.76	69.93 ± 11.55	*p* = 0.04	Student’s t-test
Height (cm)	157.25 ± 5.78	158.79 ± 6.06	*p* = 0.49	Student’s t-test
BMI (kg/m^2^)	24.33 ± 3.38	27.69 ± 4.06	*p* = 0.02	Student’s t-test
Cholesterol (mg/dL)	203.33 ± 43.66	199.68 ± 21.22	*p* = 0.76	Student’s t-test
Triglyceride (mg/dL)	128.17 ± 89.37	173.97 ± 70.48	*p* = 0.01	Wilcoxon rank-sum test
HDL (mg/dL)	55.38 ± 12.46	47.81 ± 9.09	*p* = 0.01	Wilcoxon rank-sum test
LDL (mg/dL)	132.30 ± 44.24	128.63 ± 18.70	*p* = 0.75	Student’s t-test
SBP (mm Hg)	116.69 ± 8.50	152.81 ± 15.85	*p* < 0.0001	Student’s t-test
DBP (mm Hg)	77.97 ± 10.30	104.05 ± 10.08	*p* < 0.0001	Student’s t-test

### Effect size calculation

To determine the number of subjects required for this study, the effect size was calculated using the pwr.2p.test function of the pwr package (version 1.3-0) (Champely, 2020) in R version 4.4.3 (R Core Team, 2025) to compare two independent proportions (HT *vs*. NHT groups). Based on the expectation of a substantial difference in outcomes between the groups, a large effect size (h = 0.8) was assumed. A two-sided significance level (alpha) of 0.05 and statistical power of 0.80 were specified. The required sample size was calculated as 25 participants per group. However, due to recruitment limitations based on voluntary participation through community clinics within a single district, the study included only 31 participants (NHT, *n* = 12; HT, *n* = 19), which fell short of the calculated requirement.

### Fecal sample collection and bacterial genomic DNA extraction

Fecal sample collection and transportation processes were modified from those used by Gruneck et al. (2022). Briefly, fresh feces were collected using a fecal collection sheet placed in a flush toilet. Approximately 0.5 g of the fecal sample was collected using a small plastic spoon and transferred into a 30 mL sterile container. The containers were stored in a 10 kg dry ice box prepared for transportation. The collected samples were immediately transferred to the Department of Biochemistry, Phramongkutklao College of Medicine (location: 13°46′10.3″N 100°31′48.7″E) and frozen at −80 °C. Bacterial DNA was extracted from the fecal samples using the innuPREP Stool DNA Kit (Analytik Jena Biometra, Jena, Germany) following the manufacturer’s guidelines. DNA concentration and purity were evaluated using 2% (w/v) agarose gel electrophoresis. Spectrophotometry was applied to determine the DNA concentration (ng/µL) using the Take3 Microvolume Plate (Agilent Biotek, Winooski, VT, USA). The total DNA per gram of fecal wet weight was calculated and recorded. The DNA samples were stored at −20 °C until use.

### *16S rRNA* gene amplicon sequencing and data processing

The V3–V4 hypervariable region of the *16S rRNA* gene was amplified using a two-step polymerase chain reaction (PCR) method with the specific primers 341F (5′-CCTAYGGGRBGCASCAG-3′) and 806R (5′-GGACTACNNGGGTATCTAAT-3′) (Klindworth et al., 2013) for the first step, followed by index-barcode primers for the second step. The first PCR was performed using Phusion High-Fidelity PCR Master Mix (New England Biolabs, Ipswich, MA, USA) under the following conditions: an initial denaturation at 94 °C for 3 min, followed by 25 cycles of denaturation at 94 °C for 30 s, annealing at 50 °C for 30 s, and extension at 72 °C for 30 s, with a final extension at 72 °C for 10 min. The second PCR used the same master mix and cycling conditions, incorporating index-barcode primers, but with the number of cycles reduced to 10. PCR amplicons with index sequences were sequenced using a MiSeq Reagent Kit v3 (Illumina, San Diego, CA, USA) at NovoGene (Beijing, China). Amplicon sequence variants (ASVs) were assembled, trimmed, and denoised based on a quality score higher than 20 using the DADA2 pipeline (Callahan et al., 2016) in QIIME2 version 2024.5. ASVs were taxonomically classified against SILVA 138.1 SSU Ref NR 99 (Quast et al., 2013). To ensure data representativeness, a commonly used threshold was applied. Taxa with a relative abundance greater than 0.1% were classified as major-abundance taxa, whereas taxa with a relative abundance less than 0.1% were considered minor-abundance taxa.

### Microbial diversity and composition analyses

Alpha and beta diversities were analyzed using QIIME2. The alpha-diversity index values, number of observed features, Shannon index value, Faith’s phylogenetic diversity (PD) value, and Pielou evenness value were determined at a sequence depth of 14,720 reads per sample using the QIIME diversity alpha-group-significance script. Group comparisons for alpha diversity were performed using the Wilcoxon rank-sum test (*p* < 0.05) and visualized with ggplot2 (version 3.5.2) (Wickham, 2016). Beta diversity, based on Jaccard and Bray–Curtis dissimilarity, weighted UniFrac distance, and unweighted UniFrac distance, was determined using the QIIME diversity beta-group significance script. Permutational multivariate analysis of variance (PERMANOVA) with 999 permutations was used to investigate the statistical differences between the HT and NHT groups. Gut microbiota abundance was compared using Wilcoxon rank-sum tests (*p* < 0.05) on rarefied tables, using Wilcoxon test function in R.

### Linear discriminant analysis effect size (LEfSe)

LEfSe was calculated using Galaxy software (version 1.0; http://galaxy.biobakery.org/; accessed December 6, 2023) (Segata et al., 2011). Bacterial composition data were obtained for all samples from the phylum to species levels (Table S2), and the taxa were subjected to linear discriminant analysis (LDA) using a one-against-all strategy. Taxa showing an LDA score higher than 2.0, with a *p*-value less than 0.05 were selected as enriched taxa for each group.

### Participant characteristics and dietary consumption analysis

All downstream analyses were conducted in R. Participant characteristics, including age, gender, weight, height, body mass index (BMI), cholesterol, triglyceride, high density lipoprotein (HDL), low density lipoprotein (LDL), systolic blood pressure (SBP), and diastolic blood pressure (DBP) were compared between NHT and HT groups. Statistical analyses accounted for normality and homogeneity of variance, with *p*-values. Dietary consumption was compared using the Wilcoxon rank-sum test (*p* < 0.05). Multiple correspondence analysis (MCA) was used to visualize dietary consumption profiles in NHT and HT groups, implemented using the FactoMineR (version 2.11) and factoextra (version 1.0.7) packages. Consumption frequencies were categorized into five levels for MCA: daily (7), 5–6 times/week, 3–4 times/week, 1–2 times/week, and less than once per week (0). The Hierarchical All-against-All Association (HAllA) method (Ghazi et al., 2022), implemented in Python (version 3.10.3), was used to identify associations between participant characteristics, dietary consumption, and gut microbiome at phylum and genus levels.

### Multivariate statistical analysis

Multiple Factor Analysis (MFA) was performed to integrate anthropometric data, dietary consumption, and gut microbiome at the genus level, allowing visualization of features contributing to overall variation. All numeric variables, including age and blood profiles, were scaled (“s”). Dietary data were treated as frequency blocks (“f”). Genus count data were transformed using the centered log-ratio (CLR) method. Group, gender, and BMI categories were included as supplementary categorical variables. MFA was conducted using the FactoMineR package (version 2.11) (Lê, Josse & Husson, 2008), and variable profiles were visualized in the MFA space using the factoextra package (version 1.0.7) (Kassambara & Mundt, 2020).

### Functional profile analysis

Gut microbiota functional profiles were predicted using PICRUSt2 (version 2.5.3) (Douglas et al., 2020). Predicted pathways (PWs) were mapped to pathway information retrieved from http://vm-trypanocyc.toulouse.inra.fr/query.shtml (accessed August 27, 2025) to obtain pathway descriptions, parent-class, superpathways, subpathways, and taxonomic ranges. Annotated KEGG genes (K numbers) were mapped against the KEGG database (https://rest.kegg.jp/get/br:ko00001, accessed August 27, 2025) to obtain pathway-level functional annotations. Differentially abundant PWs and KOs between NHT and HT groups were identified using the ALDEx2 package (version 1.38.0), with significance determined by within-instance Wilcoxon *p*-values (*wi.ep* < 0.05) and adjusted Wilcoxon *p*-values (*wi.eBH* < 0.05). Top abundant PWs and KOs were visualized using the ComplexHeatmap package (version 2.22.0), with hierarchical clustering of rows and columns based on Spearman correlation. Associations between gut microbiota and predicted functions (PWs and KOs) were assessed using the HAllA method, implemented in Python (version 3.11.12). Genus counts and predicted functional data were subjected to centered log-ratio (CLR) transformation prior to HAllA analysis. Strong associations were filtered using a Spearman correlation coefficient threshold of |*r*| ≥ 0.8 and *q* < 0.05 for presentation in the results.

### Data availability statement

Raw sequence data from this study were deposited in the National Center for Biotechnology Information (NCBI) database and are available in the Sequence Read Archive (SRA): PRJNA1202055 (BioSample accession numbers: SAMN45949641–SAMN45949671).

## Results

### Anthropometric, clinical, and dietary profiles between NHT and HT groups

Significant differences were observed in age, triglyceride, HDL, SBP, and DBP between NHT and HT groups (Table 1). All variables were higher in HT compared to NHT, except for HDL, which was lower in HT. Comparison of dietary profiles revealed nominal differences in the consumption of milk, vegetables, and beef between NHT and HT at *p* < 0.05 (Table S3). MCA of categorical dietary data indicated that HT and NHT groups were weakly distinguished by diet (cos^2^ = 0.06 on Dim 1), implying that dietary variation contributed minimally to the overall group separation in the MCA space (Figs. S1A, S1B). HAllA analysis of associations between dietary data and gut microbiota at the genus level detected few significant associations (*q* < 0.05). Only one strong association was observed between brown rice and *Plesiomonas* in HT (Table S4) (Fig. S1C), whereas associations between pickled fruit and *Izemoplasmatales* and between tea and coffee and *Mitsuokella* were detected in NHT (Table S5) (Fig. S1D). These results indicated limited differences in dietary consumption profiles between HT and NHT, with minimal associations between diet and gut microbiota.

### Gut microbiome diversity between the NHT and HT groups

The gut bacterial composition was profiled by sequencing the *16S rRNA* V3–V4 region. Eventually, 4,059,506 high-quality sequences corresponding to 76,329 ± 31,507 reads per sample were clustered into 3,126 ASVs. These were assigned to 2 domains, 16 phyla, 26 classes, 68 orders, 127 families, 339 genera, and 696 species. The ASV counts for each sample are tabulated in Table S6, along with the taxonomic information.

**Figure 1:**
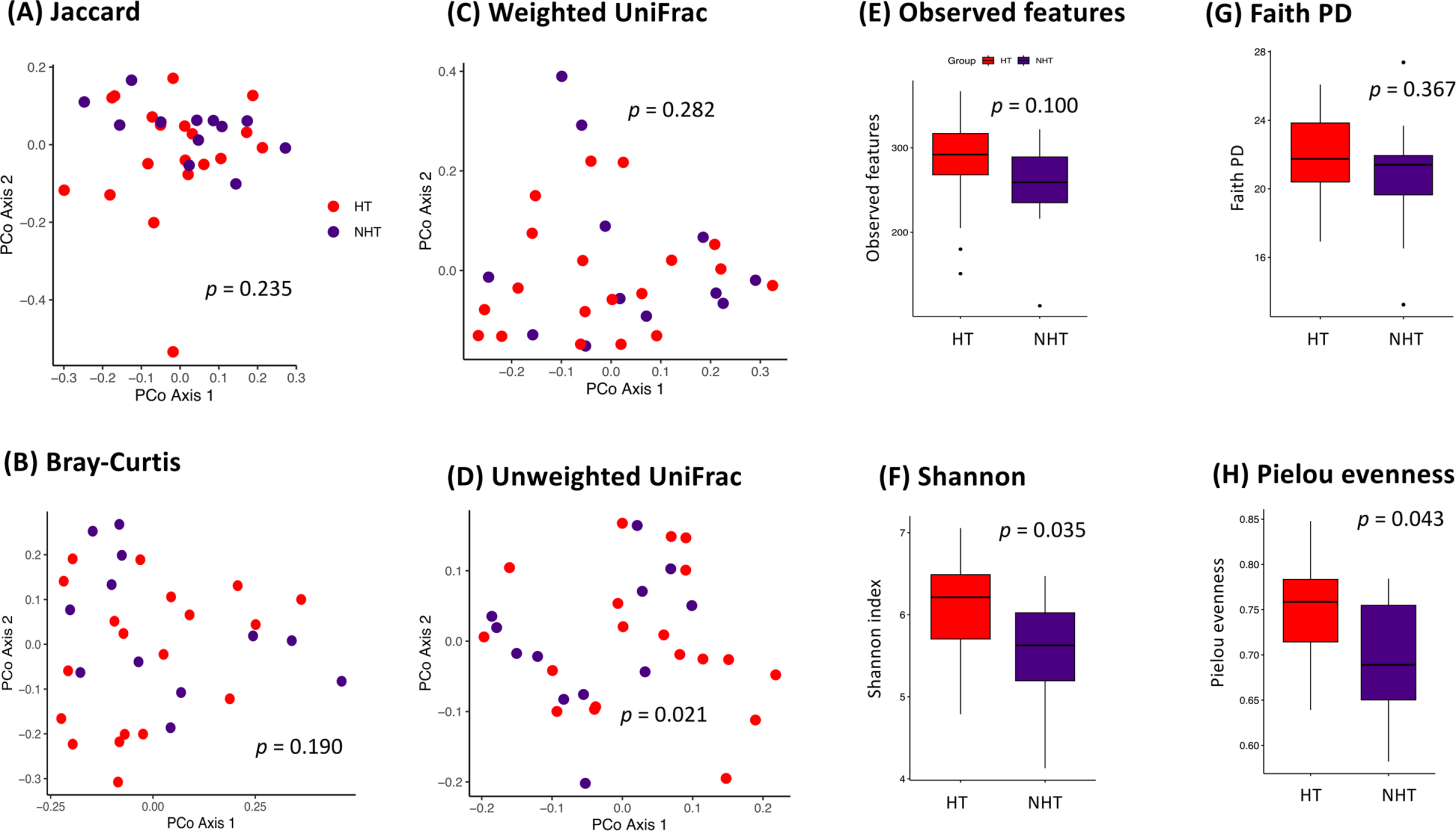
Alpha and beta diversity analyses of the microbial community composition. Beta diversity indices included (A) Jaccard, (B) Bray–Curtis dissimilarity, (C) weighted UniFrac distance, and (D) unweighted UniFrac distance. The red sphere represents the hypertensive (HT) group and the green sphere represents the non-hypertensive (NHT) group. The statistical significance of the difference between the HT and NHT groups was assessed using PERANOVA (pairwise adonis). Alpha diversity indices included (E) observed features, (F) Shannon, (G) Faith phylogenetic diversity (PD), and (H) Pielou evenness. The statistical significance of the difference between the HT and NHT groups was assessed using the Wilcoxon rank-sum test.

Diversity in the gut microbial communities was investigated using alpha and beta diversity analyses. Based on beta diversity, four distance matrices and discreteness between groups were examined using permutation analysis. Only the unweighted UniFrac distance showed a significant difference between the HT and NHT groups (*p* = 0.021) (Fig. 1D), whereas there were no significant differences in Jaccard, Bray–Curtis, or weighted UniFrac distances (*p* > 0.05) (Figs. 1A–1C). Because the unweighted UniFrac distance has been used to analyze microbial diversity based on the presence or absence of taxa (Chen et al., 2012), these results suggested that the composition of the microbial communities between the two groups differs, but the relative abundance of taxa within those communities is similar. Alpha diversity analysis indicated the complexity of the microbial communities. The observed features and Faith’s PD showed that the gut microbiome diversity did not differ significantly between the two groups (Figs. 1E and 1G). In contrast, the Shannon and Pielou indices indicated that gut microbiome richness and evenness were significantly higher in the HT group than in the NHT group (*p* < 0.05) (Figs. 1F and 1H). These results suggested that both groups had a similar range of microbial taxa and evolutionary diversity, but the distribution of these taxa differed significantly between the two groups. However, the HT group had a local alteration, unlike dysbiosis, in the gut microbiota, since there is not much information about the cutoff for alpha diversity in gut dysbiosis. Further research is needed to determine whether this constitutes a state of dysbiosis defined by significant functional impairments and clinical manifestations.

**Figure 2:**
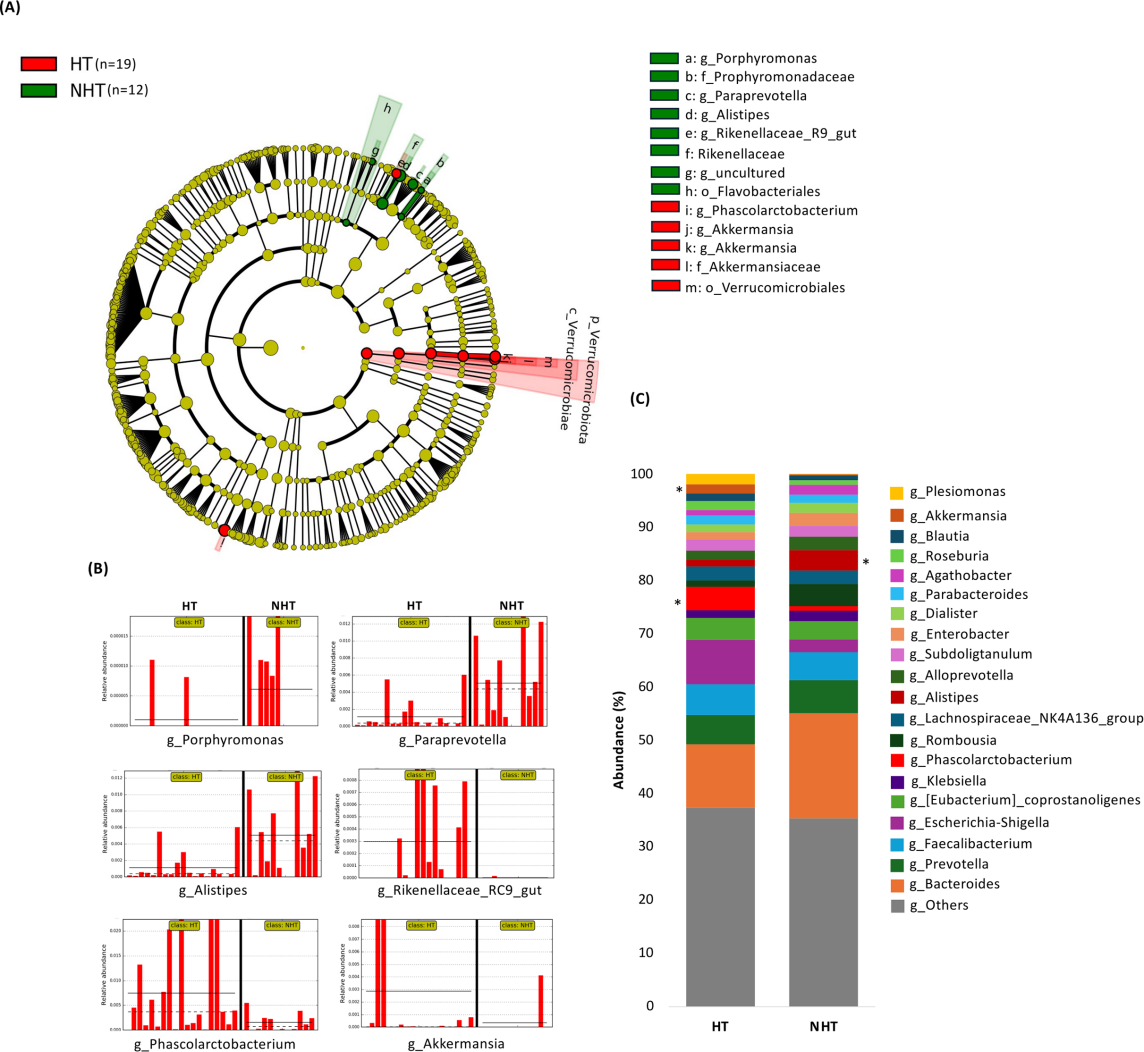
Comparison of bacterial features between the hypertensive (red; HT) and non-hypertensive (green; NHT) groups. (A) Linear discriminant analysis of effect size (LEfSe) analysis was used to compare the gut bacterial composition at each taxonomic rank between the HT and NHT groups. The Wilcoxon rank-sum test was used to calculate the linear discriminant analysis (LDA) scores. Taxonomic groups with LDA > 2.0 and *p* < 0.05 are highlighted by the indicated color on the cladogram. (B) Bar charts of the abundance of genera that differed significantly between the HT and NHT groups in the LEfSe analysis. (C) Comparison of the relative abundance of the top 20 genera in the HT and NHT groups. The abundance of each genus is shown in the stacked bar charts. The asterisk * indicates higher values in the relevant group, approaching significance (*p* < 0.05).

LEfSe analysis was performed to investigate significant differences in gut microbiome composition at different taxonomic levels between the NHT and HT groups. The cladogram showed slight differences in taxonomic diversity between the two groups (Fig. 2A). The relative abundances of six genera were significantly different between the groups. The genera *Rikenellaceae_RC9_gut*, *Phascolarctobacterium*, and *Akkermansia* differed significantly in the HT group, whereas *Porphyromonas*, *Paraprevotella*, and *Alistipes* differed significantly in the NHT group. The relative abundance of these genera in each subject is shown in Fig. 2B. Notably, *Rikenellaceae_RC9_gut*, *Porphyromonas*, and *Paraprevotella* were minor genera, with abundances less than 0.1%. The substantial contribution of *Akkermansia* to the average abundance in the HT group was driven by exceptionally high levels in only two subjects, suggesting potential heterogeneity within the HT group. A comparison of the relative abundances of the top 20 genera in the HT and NHT groups is shown in Fig. 2C to corroborate the results.

### Correlation between gut microbiome composition and host parameters associated with hypertension

**Figure 3:**
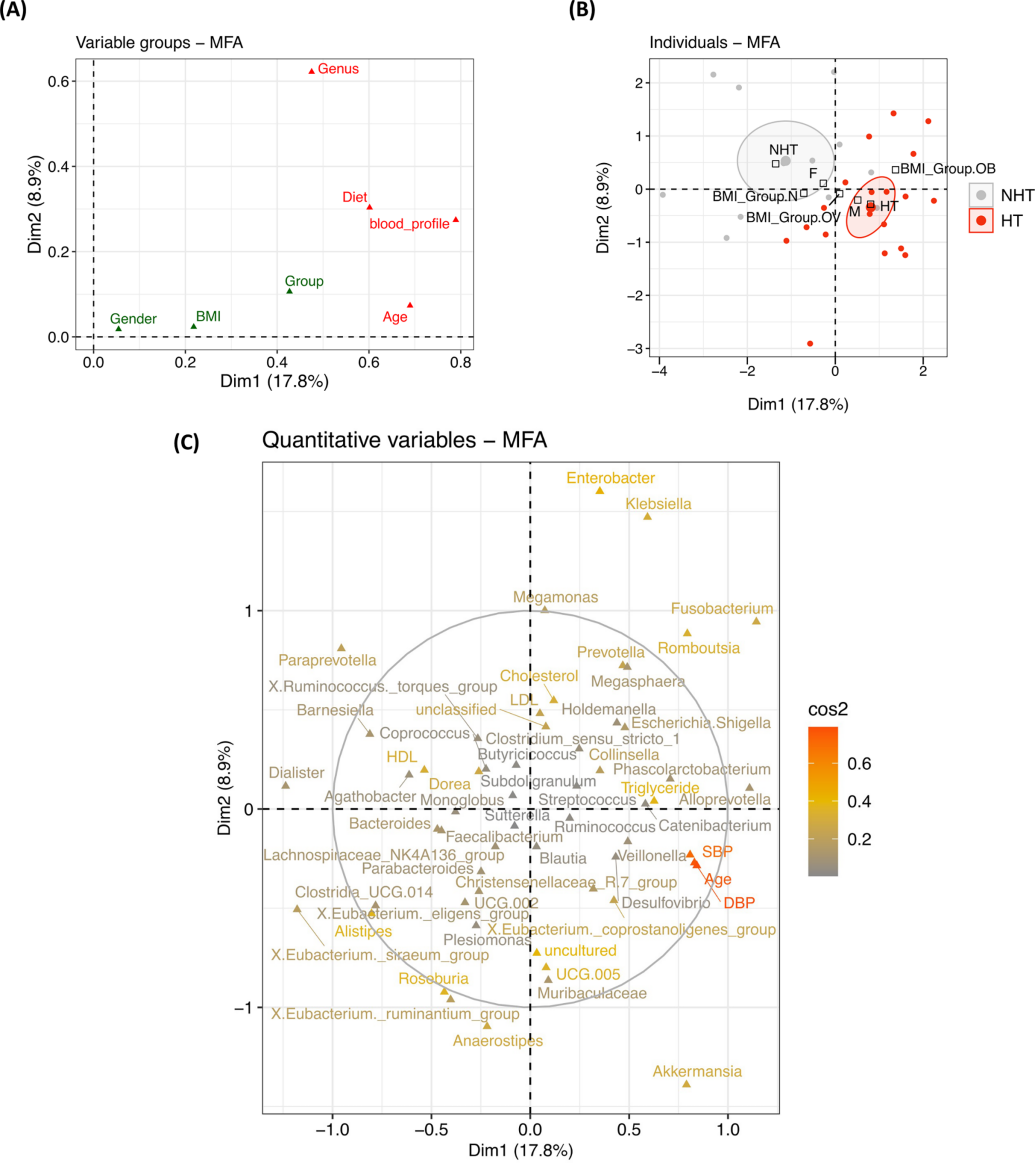
Multiple factor analysis (MFA) integrating host parameters and gut microbiota between NHT and HT groups. (A) Correlation plot between variable groups and dimensions. (B) Individual factor map showing clustering of samples by group, with 95% confidence ellipses displayed for dimensions 1 and 2. Supplementary qualitative variable categories are shown in black. (C) Quantitative variable plot displaying cos^2^ values, which indicate the quality of representation on the factor map. A variable is considered well represented by two dimensions when the sum of its cos^2^ values across these dimensions approaches one. cos^2^ values range from high (red) to low (grey). Groups: NHT = 12, HT = 19.

Integration of clinical and anthropometric data, diet, and gut microbiome composition (top 50 genera) using multiple factor analysis (MFA) explained 17.8% and 8.9% of the variance on Dimension 1 (Dim 1) and Dimension 2 (Dim 2), respectively (Fig. 3, Data S2). Dim 1 predominantly captured individual variation associated with group separation, with age (cos^2^ = 0.47) and blood profiles (cos^2^ = 0.36) as the main contributors. DBP (*r* = 0.84, *p* < 0.0001), age (*r* = 0.84, *p* < 0.0001), and SBP (*r* = 0.80, *p* < 0.0001) were highly correlated with Dim 1, and their profiles differed between NHT (coordinate = −1.08, *p* < 0.0001) and HT groups (coordinate = 1.08, *p* < 0.0001). Dietary variables showed moderate correlations with Dim 1, including milk (*r* = −0.59, *p* < 0.001) and brown rice (*r* = −0.52, *p* < 0.01). Variation in other dimensions (Dim 2 to Dim 4) was observed; however, these differences were not associated with group separation. Overall, MFA indicated that age and blood profiles were the primary drivers of individual variation between NHT and HT groups, diet was a secondary contributor, and gut microbiota composition had minimal influence.

**Figure 4:**
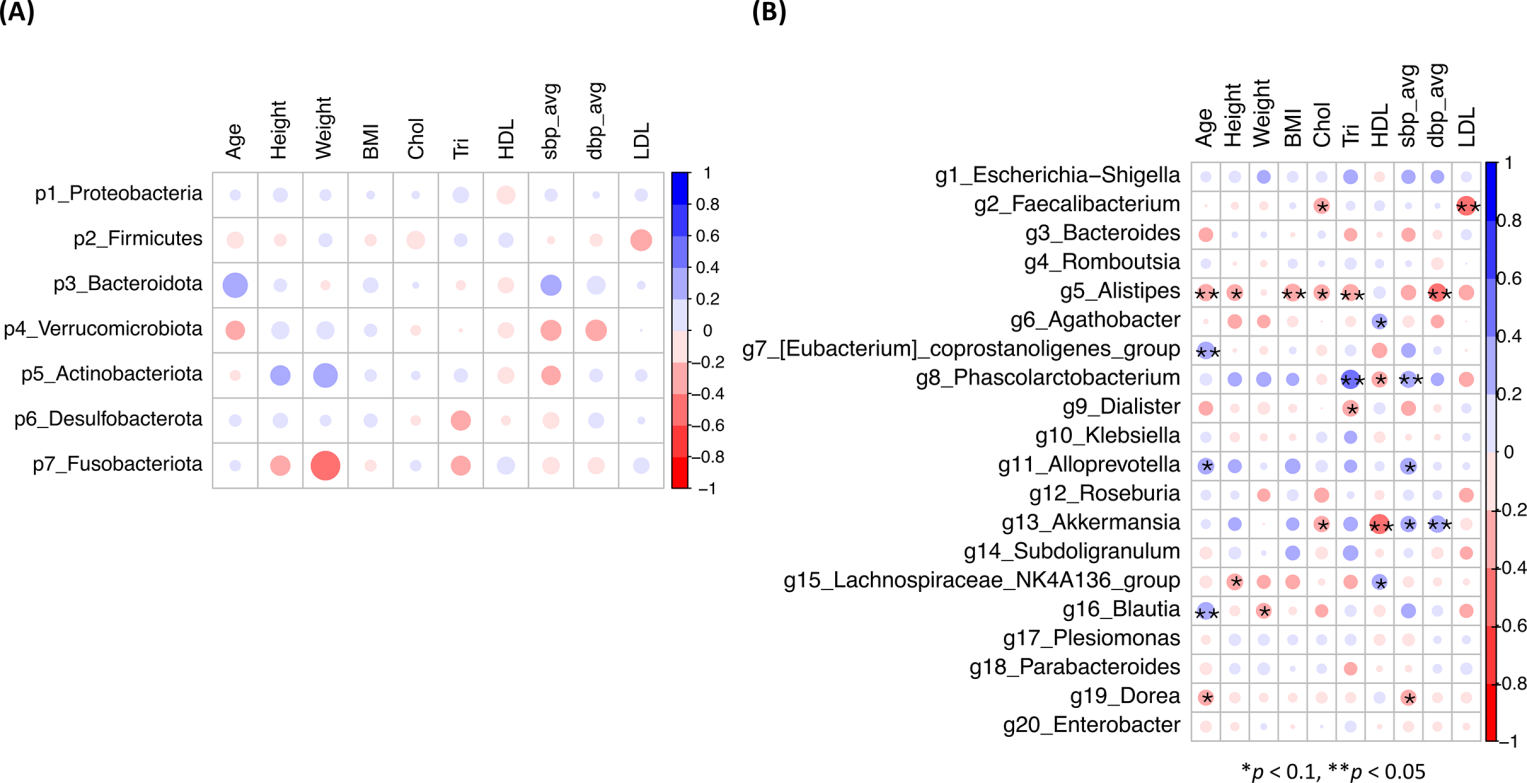
Associations between major gut bacteria and host parameters analyzed by Hierarchical All-against-All Association (HAllA). (A) Phylum level. (B) Genus level. Blue indicates positive associations, and red indicates negative associations. The asterisks * and ** indicate significant correlations, approaching at *p* < 0.1 and *p* < 0.05, respectively.

Bacterial taxa at the phylum (p1–p7) and genus (g1–g66) levels, with an abundance greater than 0.1%, were subjected to the HAllA analysis. The correlations between SBP, DBP, cholesterol, triglyceride, age, and BMI as indices associated with hypertension were analyzed for each sample donor (Tables S7, S8). The result showed that no significant correlations between phyla and these parameters were found (Fig. 4A). At the genus level, the abundances of the key genera, *Alistipes* (g5) and *Phascolarctobacterium* (g8), were significantly correlated with these parameters, but they showed likely opposite relationships (Fig. 4B). The abundance of *Alistipes* (g5), which was primarily enriched in the NHT group, exhibited significant negative correlations with cholesterol (*p* < 0.1), age, BMI, triglyceride, and DBP (*p* < 0.05) (Fig. 4B). Conversely, the abundance of *Phascolarctobacterium* (g8), which was predominantly enriched in the HT group, showed significant positive correlations with SBP and triglyceride levels (*p* < 0.05), but negative correlation with HDL levels (*p* < 0.1). The abundance of *Akkermansia* (g13) showed significant positive correlations with SBP (*p* < 0.1) and DBP (*p* < 0.05), but significant negative correlations with cholesterol levels (*p* < 0.1) and HDL levels (*p* < 0.05). However, it should be noted that elevated abundance of *Akkermansia* (g13) was primarily driven by two subjects in the HT group, as described in the LEfSe results.

### Differences in association patterns between gut microbiome and predicted functions (PWs and KOs) in NHT and HT

**Figure 5:**
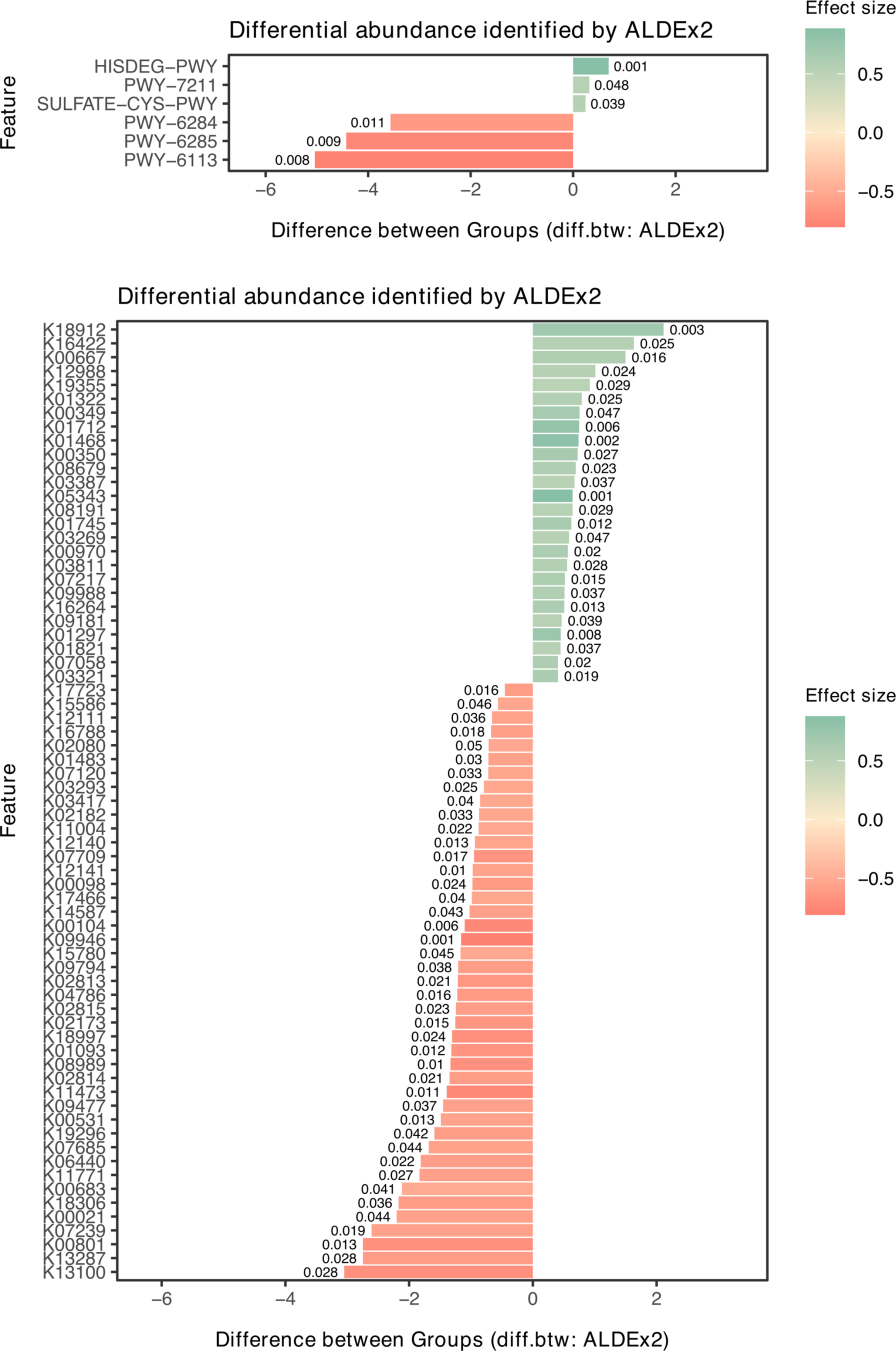
Differential functional abundance between NHT and HT groups determined by ALDEx 2 analysis. (A) Predicted functional pathways (PWs) and (B) KEGG Orthologs (KOs). Green bars indicate higher abundance in the HT group, while red bars indicate lower abundance. Effect size thresholds: ≥ 0.5 (small), 1.0–1.5 (moderate), and > 1.5 (large). A larger effect size reflects stronger between-group differences relative to within-group variation. Bar labels represent *p*-values (wi.ep < 0.05). Groups: NHT = 12, HT = 19. The HT group exhibits a distinct functional profile characterized by reduction in fatty acid biosynthesis pathways (PWY-6284, PWY-6285, PWY-6113), suggesting a shift in metabolic potential.

A total of 451 predicted pathways (PWs) and 9,843 KEGG orthologs (KOs) were identified (Data S3). The most abundant features across groups were NONOXIPENT-PWY (pentose phosphate pathway, non-oxidative branch) and K03088 (rpoE; RNA polymerase sigma-70 factor, ECF subfamily) (Fig. S2). Among these, six PWs and 69 KOs were differentially abundant between NHT and HT at *wi.ep* < 0.05 using ALDEx2. For example, pathways related to unsaturated fatty acid biosynthesis (PWY-6284) and fatty acid biosynthesis (PWY-6285, PWY-6113) were less abundant in HT compared with NHT (Fig. 5A). At the KO level, K13100 (CWC22; pre-mRNA-splicing factor CWC22) and K18912 (egtB; gamma-glutamyl hercynylcysteine S-oxide synthase) showed the largest differences between groups, with lower and higher abundance in HT, respectively (Fig. 5B). However, none of these differences remained statistically significant after multiple-testing correction (*wi.eBH* < 0.05).

**Figure 6:**
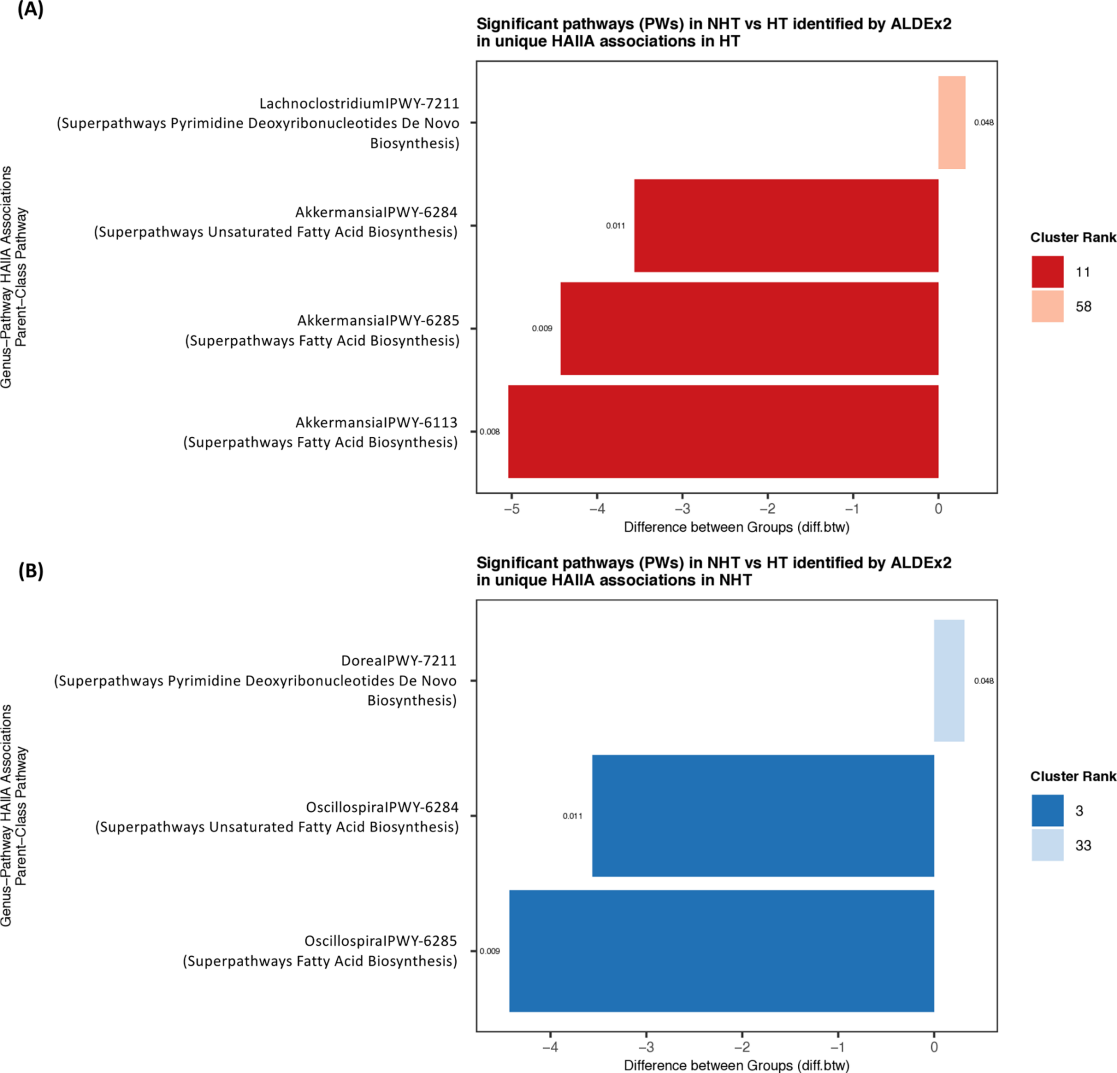
Significant predicted functions associated with gut microbiota. Significant (A) predicted functional pathways (PWs) and (B) KEGG Orthologs (KOs) differing between NHT and HT groups based on ALDEx2 analysis at the genus level. Bar colors represent cluster ranks identified by HAllA. Positive differences indicate higher abundance, while negative differences indicate lower abundance in the HT group relative to NHT. Groups: NHT = 12; HT = 19. Significant (A) predicted functional pathways (PWs) and (B) KEGG Orthologs (KOs) differing between NHT and HT groups based on ALDEx2 analysis at the genus level. Bar colors represent cluster ranks identified by HAllA. Positive differences indicate higher abundance, while negative differences indicate lower abundance in the HT group relative to NHT. Groups: NHT = 12; HT = 19.

**Figure 7:**
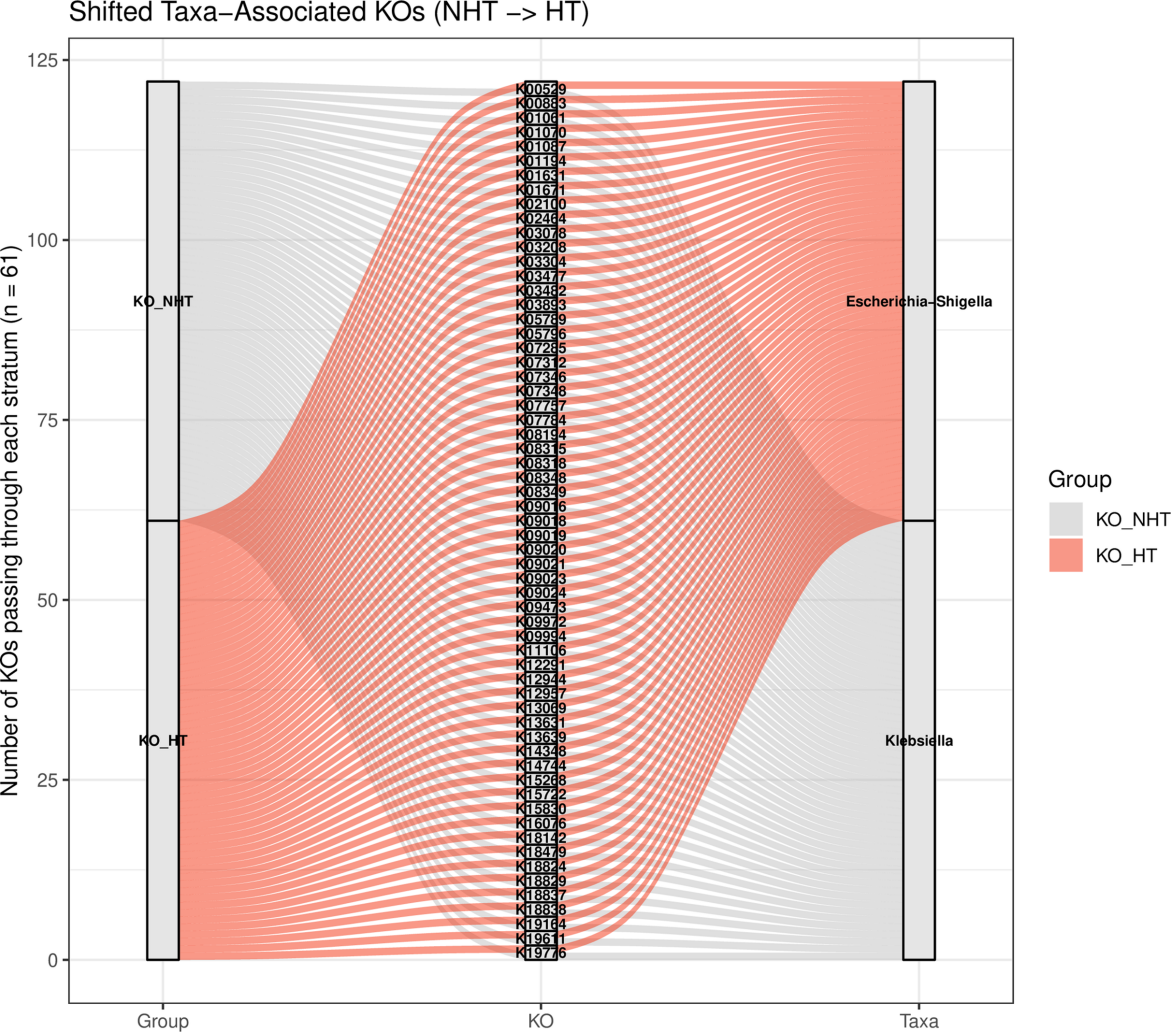
Alluvial plot illustrating shifts in microbe-function associations identified by HAllA. The flow connects metabolic pathways (left) to specific bacterial taxa (right) in NHT (grey) and HT (orange) groups. Each stratum represents a single KO, highlighting how the same KOs are linked to different taxa across the two groups. Groups: NHT = 12; HT = 19. Functional KOs shift their primary taxonomic associations from *Klebsiella* in NHT to *Escherichia-Shigella* in HT subjects, indicating a potential reorganization of the microbial ecosystem .

Associations between gut microbiota and predicted functions were further examined using HAllA (Data S3). Filtering at |*r*| ≥ 0.8 and *q* < 0.05 revealed shifts in microbial–functional association patterns between groups. The number of significant strong and unique associations was higher in HT than in NHT, while relatively few associations were shared (Fig. S3). In HT, gut microbiome showed positive associations with both PWs and KOs. *Lachnoclostridium* formed the largest number of PW associations in HT, whereas *Klebsiella* was dominant in NHT. In HT, pathways from parent classes such as aromatic compound degradation, glycogen and starch biosynthesis, and autotrophic CO_2_ fixation were strongly associated with gut microbiota (Fig. S4). Associations of PWY-6284 and PWY-6285 also shifted from *Oscillospira* in NHT to *Akkermansia* in HT (Fig. 6). At the KO level, dominant associations shifted from *Klebsiella* in NHT to *Blautia* in HT, with the number of interactions reduced by more than half. Some genus–KO clusters transitioned entirely to *Blautia*–KO associations in HT (Table 2). *Escherichia*–*Shigella* emerged as the third most abundant KO-associated genus in HT, replacing *Klebsiella* in NHT, and was largely linked to KOs within the category 09183 Protein families: signaling and cellular processes (Fig. 7). None of these shifted associations overlapped with KOs identified as differentially abundant by ALDEx2 (*wi.ep* < 0.05). Moreover, enrichment patterns also differed between groups. KEGG level 2 pathways enriched in unique associations included 09183 Protein families: signaling and cellular processes in NHT and 09152 Endocrine system in HT (Fig. S5). Together, these results highlight that hypertension status may be associated with shifts in gut microbiota–function associations at both the PW and KO levels.

**Table 2:** Shifts in microbial–KO associations between NHT and HT groups.

KO	NHT	HT	KO description	KEGG pathway level 2	Cluster rank
K00620	*Oscillibacter*	*Blautia*	argJ; glutamate N-acetyltransferase/amino-acid N-acetyltransferase	09105 Amino acid metabolism	419
K02006	*UCG-002*	*Blautia*	cbiO; cobalt/nickel transport system ATP-binding protein	09131 Membrane transport; 09183 Protein families: signaling and cellular processes	298
K02008	*UCG-002*	*Blautia*	cbiQ; cobalt/nickel transport system permease protein	09131 Membrane transport; 09183 Protein families: signaling and cellular processes	508
K02049	*Ruminococcus*	*Blautia*	ABC.SN.A; NitT/TauT family transport system ATP-binding protein	09183 Protein families: signaling and cellular processes	564
K02050	*Ruminococcus*	*Blautia*	ABC.SN.P; NitT/TauT family transport system permease protein	09183 Protein families: signaling and cellular processes	628
K03700	*Coprococcus; Oscillibacter*	*Blautia*	recU; recombination protein U	09182 Protein families: genetic information processing	563
K07045	*Coprococcus*	*Blautia*	K07045; uncharacterized protein	09194 Poorly characterized	468
K09825	*Holdemanella*	*Blautia*	perR; Fur family transcriptional regulator, peroxide stress response regulator	09182 Protein families: genetic information processing	468
K10121	*Dorea*	*Blautia*	msmF; fructooligosaccharide transport system permease protein	09183 Protein families: signaling and cellular processes	492
K15634	*Roseburia*	*Blautia*	gpmB; 2,3-bisphosphoglycerate-dependent phosphoglycerate mutase	09101 Carbohydrate metabolism; 09102 Energy metabolism; 09105 Amino acid metabolism	958

## Discussion

Although the relationship between the gut microbiota and cardiovascular diseases, including hypertension, has been increasingly recognized, its relevance within the context of Thailand’s evolving dietary landscape remains insufficiently characterized. In this exploratory, community-based study, we sought to profile the gut microbial community and examine its potential associations with dietary patterns and hypertension-related host parameters among Thai individuals. Given the multidimensional and compositional nature of microbiome data, multivariate and compositional analytical frameworks were employed to identify coordinated variation across microbial, dietary, and clinical domains. These analyses were intended to detect structured patterns rather than establish causal inference; therefore, considering the modest cohort size, the observed associations should be interpreted as hypothesis-generating and warrant confirmation in larger, independent populations.

The gut microbial diversity between the hypertensive and non-hypertensive groups exhibited a complex pattern, with inconsistent trends in the alpha- and beta-diversity index values. However, these indices suggested significant differences in the microbial community composition and distribution between the two groups. LEfSe analysis further corroborated these findings and identified specific differentially abundant taxa in the HT group. While these changes indicate a local alteration in the gut microbiota, the absence of a clear cutoff for alpha diversity in gut dysbiosis precludes definitive conclusions regarding the extent of dysbiosis. Further investigations are required to determine the functional and clinical significance of these microbial alterations.

The gut microbiome of the HT group exhibited a distinct profile with an increased abundance of *Phascolarctobacterium* and a decreased abundance of *Alistipes*. This finding aligns with the findings of a study by Ding et al. (2023), who reported higher *P. faecium* and lower *A. putredinis* levels in HT patients without T2D. The abundance of *Phascolarctobacterium* positively correlated with cholesterol and SBP levels. It is a gram-negative bacterium in the phylum Firmicutes, which produces acetate and propionate, and has a complex association with health. Although this bacterium shows promise as a biomarker for early-stage T2D (Li et al., 2022b; Li, Hu & Xiong, 2023), its role in hypertensive heart failure in animal models raises concerns regarding its potential contribution to cardiovascular disease (Gutiérrez-Calabrés et al., 2020; Han et al., 2021). As none of the participants had T2D, the observed microbial signatures likely reflect hypertension-specific associations rather than diabetic comorbidity, though species-level validation is required. In contrast, the abundance of *Alistipes* negatively correlated with DBP, cholesterol levels, triglyceride levels, and BMI. Although the exact mechanisms underlying this association remain unclear, *Alistipes*, a gram-negative bacterium of the phylum Bacteroidetes, has been implicated in various metabolic processes and may exert protective effects against cardiovascular diseases. *Alistipes* is involved in the metabolism of complex carbohydrates, such as plant polysaccharides (Gan, Wang & Guo, 2022). This metabolic activity can influence the production of short-chain fatty acids (SCFAs), which have been linked to various health benefits, including improved cardiovascular health (Kazemian et al., 2020; Parker et al., 2020). Previous studies have suggested that alterations in the gut microbiota, including changes in the abundance of *Alistipes*, can contribute to systemic inflammation (Parker et al., 2020). Furthermore, given its potential as an SCFA producer, a decrease in *Alistipes* abundance contributes to hepatic fibrosis by reducing the production of anti-inflammatory cytokines and weakening the suppression of TH17 cells in mice (Li et al., 2016). SCFA-producing bacteria have been known to be associated with blood pressure regulation and hypertension through various mechanisms mediated by the acids they produce. For example, SCFAs directly regulate blood pressure by binding to their receptors. Low SCFA concentrations activate GPR41/GPR43, decreasing cAMP levels *via* Gαi/Gαo, causing vasodilation and lower BP. High SCFA concentrations activate Olfr78, which increases cAMP levels *via* AC3/Golf, leading to renin release and vasoconstriction (Wu et al., 2021). In addition, SCFAs act locally to protect against blood pressure dysregulation by influencing the gut barrier and immune responses. These effects involve molecules such as FOXO3a, GLP-2, HDAC, IL, MT2, NLRP3, TH17, and TNF-α, and regulatory T cells (Wu et al., 2021). Therefore, a healthy gut microbiota, including the balanced presence of *Alistipes*, may help maintain a healthy inflammatory state, which is crucial for cardiovascular health. However, *Alistipes* has also been found to contribute to inflammation and tumorigenesis, thus defining *Alistipes* species as gut pathogens (Moschen et al., 2016). Further research is required to elucidate the specific role of *Alistipes* in hypertension and other related conditions.

Our functional analysis reveals a restructuring of the gut microbiome ecosystem in HT group, characterized by a functional dysbiosis where microbial-functional association patterns shift even in the absence of significant changes in feature abundance. The observed reduction in fatty acid biosynthesis pathways (PWY-6284, PWY-6285, PWY-6113) in the HT group suggests a diminished capacity for producing vasoprotective metabolites, which may exacerbate vascular dysfunction. This aligns with Pluznick (2017), who highlighted that microbial-derived fatty acids such as SCFAs, act as signaling molecules *via* host receptors to modulate blood pressure. Consequently, the depletion of these functional pathways likely impairs microbial-mediated pressure regulation, potentially contributing to the hypertensive phenotype observed in our cohort. Notably, the primary drivers of these functional pathways shifted from *Klebsiella* in NHT to *Lachnoclostridium* and *Blautia* in HT, alongside the emergence of *Escherichia-Shigella* in signaling and cellular processes. This taxonomic-functional replacement, exemplified by the transition of fatty acid biosynthesis associations from *Oscillospira* to *Akkermansia*, highlights a compensatory or pathological reorganization of the microbial network. The lack of balance between differentially abundant features and shifted associations further suggests that hypertension primarily influences the interaction dynamics and metabolic connectivity of the gut microbiome, rather than merely altering individual taxa, pointing toward a more complex systemic interplay in the pathogenesis of hypertension. In addition to the functional restructuring of the gut microbime, our multi-factor integration using MFA and HAllA suggests that the observed differences between the NHT and HT groups appear most strongly linked to host factors, specifically age and clinical blood profiles rather than dietary habits or overall microbial composition. The limited associations between diet and specific genera (*e.g*., brown rice and *Plesiomonas* in HT) indicate that dietary variations alone may not be the principal cause of the microbiome shifts in this study. Instead, the functional dysbiosis previously noted may be more closely linked to the physiological state of hypertension itself rather than external dietary influences, highlighting the complexity of host-microbiome interactions in hypertensive pathology.

This study has some limitations. First, the sample size was limited and imbalanced because of voluntary participation. Although the preliminary data were informative, the sample size was not sufficiently large to achieve optimal statistical power and thus, the results require confirmation in larger cohorts. Second, the imprecise recording of dietary habits limited our ability to establish causal links between diet, the gut microbiota, and hypertensive health, highlighting the need for future longitudinal studies to explore these relationships and the potential for dietary interventions. Third, the restriction of the study population to Chachoengsao Province may have limit the generalizability of the results to the broader Thai population, underscoring the importance of further research in diverse geographic locations within Thailand for a more comprehensive understanding. Finally, it is important to reiterate that the associations identified herein do not establish causation, and future research, particularly using animal models, is required to confirm these findings and elucidate the underlying mechanisms.

## Conclusions

This exploratory study suggests that hypertension in this Thai cohort was associated with functional alterations and shifts in microbial–functional interactions rather than broad changes in overall community composition. The expansion of *Phascolarctobacterium* and the depletion of *Alistipes* emerged as notable taxonomic patterns, while reduced fatty acid biosynthesis pathways may reflect altered metabolic potential. Multi-omics integration *via* MFA further indicated that age and clinical characteristics accounted for substantial inter-individual variation, with the gut microbiota showing coordinated associations within this network. Collectively, these findings highlight the relevance of microbial–host interaction dynamics beyond simple abundance changes and provide hypothesis-generating insights for future studies investigating functional microbiome targets in hypertension.

## Supplemental Information

10.7717/peerj.21135/supp-1 Supplemental Information 1 Supplemental Data.

10.7717/peerj.21135/supp-2 Supplemental Information 2 Supplemental Figures.

10.7717/peerj.21135/supp-3 Supplemental Information 3 Supplemental Tables.

10.7717/peerj.21135/supp-4 Supplemental Information 4 STROBE checklist.

## Additional Information and Declarations

### Field Study Permissions

The following information was supplied relating to field study approvals (*i.e*., approving body and any reference numbers):

Field experiments were approved by the Ethics Committees of the Institutional Review Board, Royal Thai Army Medical Department (IRBRTA0320/2024).

### DNA Deposition

The following information was supplied regarding the deposition of DNA sequences:

The sequences are available at NCBI Sequence Read Archive: PRJNA1202055.

### Data Availability

The following information was supplied regarding data availability:

The raw data is available in the Supplemental Files.

The sequences are available at NCBI Sequence Read Archive: PRJNA1202055.
