# Supplementary material for: Associations among diet, gut microbiota, and hypertension: a cross-sectional study in Thai subjects
Source: PeerJ. 2026 Apr 21;14:e21135. doi: 10.7717/peerj.21135 (PMC13108461; doi:10.7717/peerj.21135)
Supplement: Supplemental Information 1 [file peerj-14-21135-s001.zip › peerj-111937-Supplemental_Data_S1.docx]

**Associations among diet, gut microbiota, and hypertension: a cross-sectional study in Thai subjects**

Phatthanaphong Therdtatha^1^, Thanapoj Buakhao^2^, Niwed Kullawong^3,4^, Vasana Jinatham^3,5^, Thanakrit Vichasilp^2^, Jiro Nakayama^6^, Siam Popluechai^3,5^

^1^ Specialized Research in Microbiome and Metabolome for Health Laboratory, Division of Biotechnology, Faculty of Agro-Industry, Chiang Mai University, Chiang Mai, Thailand

^2^ Department of Biochemistry, Phramongkutklao College of Medicine, Bangkok, Thailand

^3^ Gut Microbiome Research Group, Mae Fah Luang University, Muang, Chiang Rai, Thailand

^4^ School of Health Science, Mae Fah Luang University, Chiang Rai, Thailand

^5^ School of Science, Mae Fah Luang University, Muang, Chiang Rai, Thailand

^6^ Laboratory of Microbial Technology, Division of Applied Molecular Microbiology and Biomass Chemistry, Department of Bioscience and Biotechnology, Faculty of Agriculture, Graduate School, Kyushu University, Fukuoka, Japan

Corresponding Author:

Siam Popluechai^3,5^

Email address: siam@mfu.ac.th

**Supplemental Data S1**

**Subject screening**

In this study, subject screening was done based on the inclusion and exclusion

criteria shown as below.

1. Subjects must not have and be diagnosed with the following conditions:

- Immunodeficiency
- Type 2 diabetes
- Recovery after surgical operation
- Systemic infections
- Systemic inflammatory disorders
- Autoimmune disorders
- Malignant cancers
- Malnutrition
- Renal insufficiency/failure
- Acute gastrointestinal diseases, for example gastroenteritis, diarrhea, and etc.

2. Subject must not be received immune-alteration medications, such as immunosuppressive drugs, anti-inflammatory drugs, and etc.

3. Subjects must not be received the following medications within six months prior to the specimen collection:

- Systemic antimicrobials (antibiotics, antifungals, antivirals or antiparasitics) via
- either intravenous (IV) or intramuscular (IM) routes
- Systemic corticosteroid
- Cytokines
- Methotrexate or immunosuppressive cytotoxic agents

4. Subjects must not be received oral antimicrobials (antibiotics, antifungals, antivirals or antiparasitics) within two months prior to the specimen collection.

5. Subjects must not consume large quantity of the commercial probiotics (> 10^8^

cfu/day) and/or prebiotics. The foods with probiotic component (e.g. yogurts, fermented food, etc.) are acceptable.

6. Subjects must not have a major surgical operation of gastrointestinal tracts within five years prior to specimen collection. Appendectomy is acceptable.

7. Subjects must not have a history of uncontrolled gastrointestinal disorders, including

- Inflammatory bowel diseases (IBD): ulcerative colitis (mild-moderate-severe) and Crohn’s disease (mild-moderate-severe)
- Irritable bowel syndrome (IBS) (moderate-severe)
- Persistent infectious gastroenteritis, colitis or gastritis
- Persistent or chronic diarrhea of unknown etiology
- Persistent or chronic constipation
- *Clostridium difficile* infection (It is not necessary to check. Only the case that subject already diagnosed.)
- Untreated Helicobacter pylori infection (It is not necessary to check. Only the case that subject already diagnosed.)

8. Subject information is required to be collected as followed:

- Age
- Gender
- Height
- Weight
- Fecal frequency and defecation condition
- Allergy
- Medication
- Diseases
- Daily exercise
- Smoking and drinking
- Food frequency questionnaire or dietary records
- Medical record
