# Supplementary material for: Associations among diet, gut microbiota, and hypertension: a cross-sectional study in Thai subjects
Source: PeerJ. 2026 Apr 21;14:e21135. doi: 10.7717/peerj.21135 (PMC13108461; doi:10.7717/peerj.21135)
Supplement: Supplemental Information 2 [file peerj-14-21135-s002.docx]

**Associations among diet, gut microbiota, and hypertension: a cross-sectional study in Thai subjects**

Phatthanaphong Therdtatha^1^, Thanapoj Buakhao^2^, Niwed Kullawong^3,4^, Vasana Jinatham^3,5^, Thanakrit Vichasilp^2^, Jiro Nakayama^6^, Siam Popluechai^3,5^

^1^ Specialized Research in Microbiome and Metabolome for Health Laboratory, Division of Biotechnology, Faculty of Agro-Industry, Chiang Mai University, Chiang Mai, Thailand

^2^ Department of Biochemistry, Phramongkutklao College of Medicine, Bangkok, Thailand

^3^ Gut Microbiome Research Group, Mae Fah Luang University, Muang, Chiang Rai, Thailand

^4^ School of Health Science, Mae Fah Luang University, Chiang Rai, Thailand

^5^ School of Science, Mae Fah Luang University, Muang, Chiang Rai, Thailand

^6^ Laboratory of Microbial Technology, Division of Applied Molecular Microbiology and Biomass Chemistry, Department of Bioscience and Biotechnology, Faculty of Agriculture, Graduate School, Kyushu University, Fukuoka, Japan

Corresponding Author:

Siam Popluechai^3,5^

Email address: siam@mfu.ac.th

**Supplemental Figures**

**Figure S1: Dietary profile and its association with gut microbiome.** (A) Multiple correspondence analysis (MCA) plot of dietary consumption categories (categorical dietary data) between NHT and HT groups. (B) Individual factor map showing group clusters with 95% confidence ellipses along dimensions 1 and 2. Heatmap of significant associations between dietary consumption (numeric frequencies) and gut microbiota at the genus level identified by HAllA. Red indicates positive associations, and blue indicates negative associations. Groups: (C) HT = 19; (D) NHT = 12.


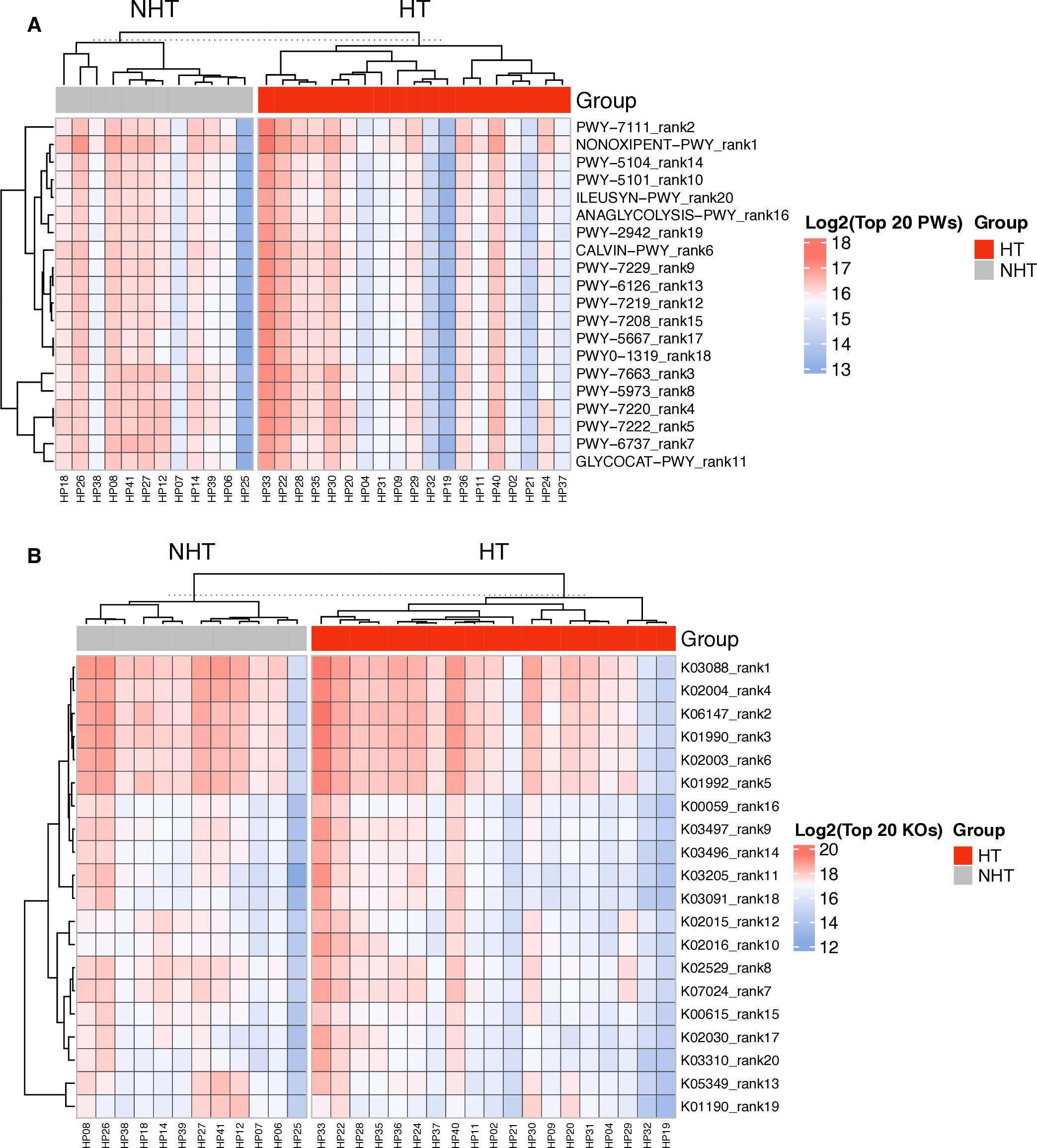


**Figure S2: Top 20 predicted functional predicted pathways (PWs) and KEGG Orthologs (KOs) in NHT and HT groups.** Heatmap showing the Log₂-transformed abundance of the top 20 (A) PWs and (B) KOs. Red indicates higher abundance, while blue indicates lower abundance. Groups: NHT = 12, HT = 19.


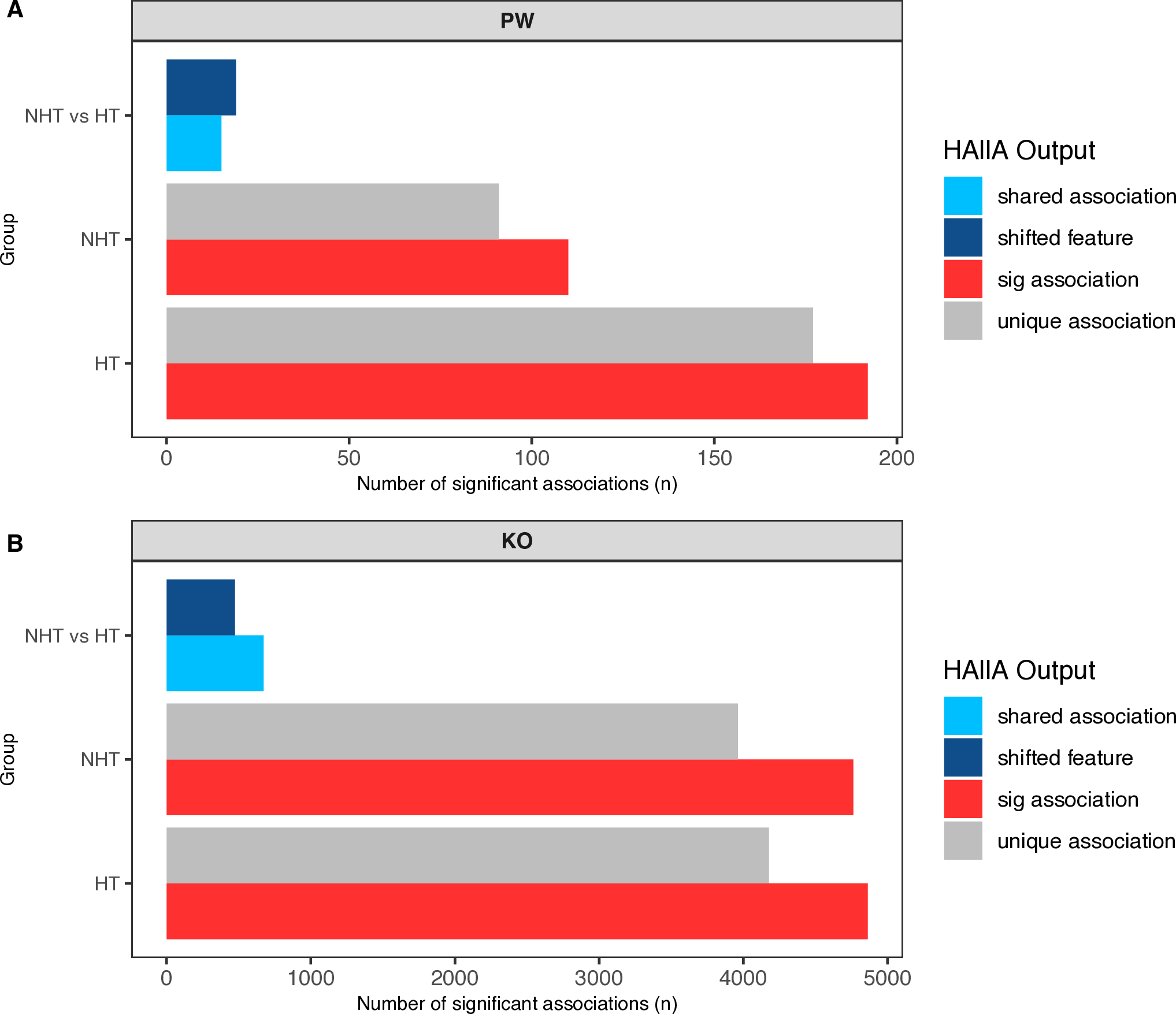


**Figure S3: Numbers of gut microbiome–functional associations identified by HAllA.** Bar plots show the number of associations categorized as significant (red), unique (grey), shared (light blue), and shifted (dark blue) between the NHT and HT groups at each functional level: (A) PW and (B) KO. Groups: NHT = 12; HT = 19.


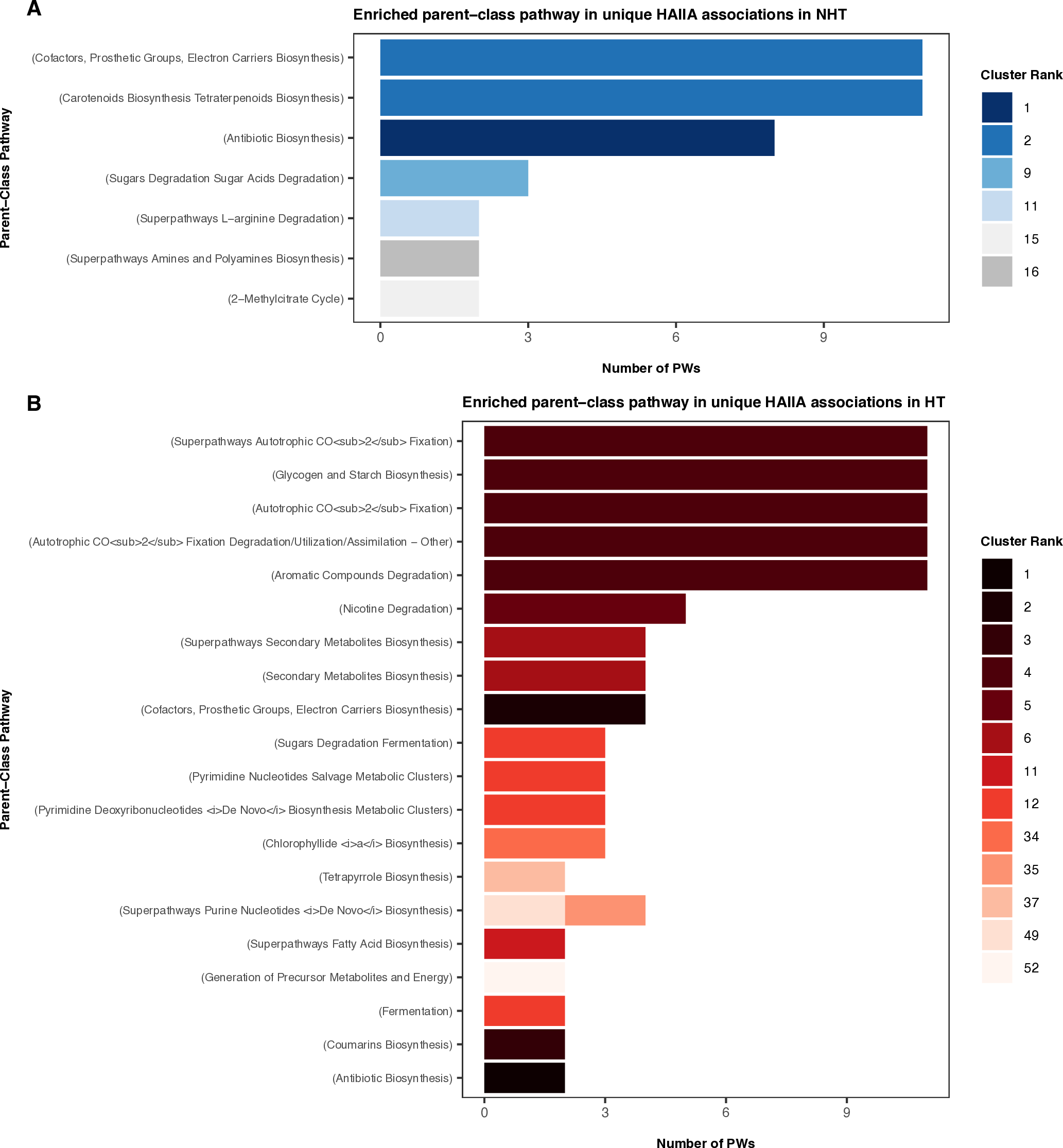


**Figure S4: Parent-class pathway distribution in unique HAllA associations**. Distributions of parent-class pathways involved in unique associations between gut microbiome and predicted functional pathways (PWs) in the (A) NHT and (B) HT groups. Bar colors represent cluster ranks identified by HAllA. Groups: NHT = 12; HT = 19.


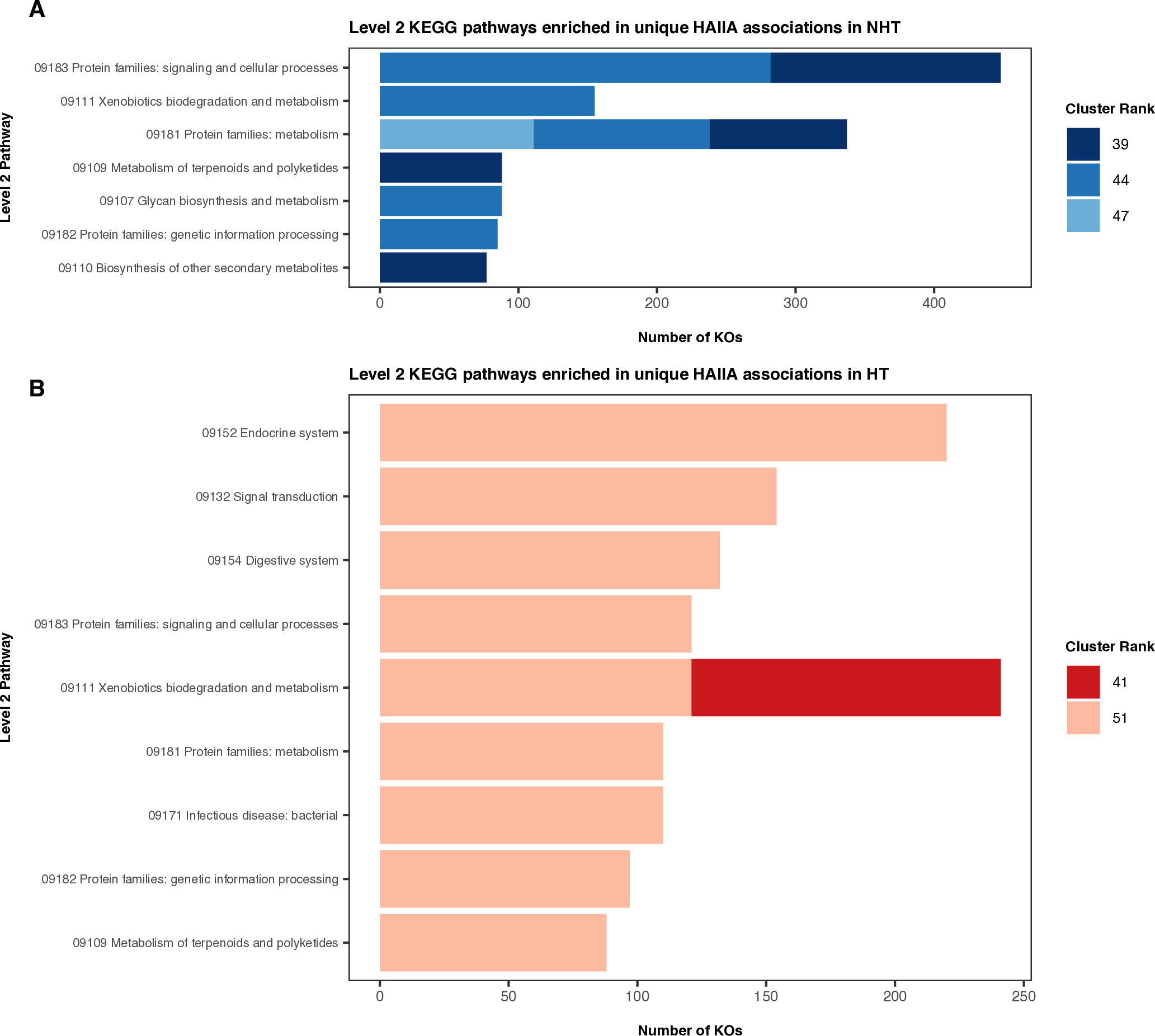


**Figure S5: Distribution of level 2 KEGG pathways in unique HAllA associations.** Distribution of level 2 KEGG pathways involved in unique associations between gut microbiome and KEGG Orthologs (KOs) in the (A) NHT and (B) HT groups. Bar colors represent cluster ranks identified by HAllA. Groups: NHT = 12; HT = 19.
